# Prevalence and risk factors for asymptomatic malaria and genotyping of glucose 6-phosphate (G6PD) deficiencies in a vivax-predominant setting, Lao PDR: implications for sub-national elimination goals

**DOI:** 10.1186/s12936-018-2367-5

**Published:** 2018-06-01

**Authors:** Andrew A. Lover, Emily Dantzer, Bouasy Hongvanthong, Keobouphaphone Chindavongsa, Susie Welty, Tania Reza, Nimol Khim, Didier Menard, Adam Bennett

**Affiliations:** 1 Malaria Elimination Initiative, Institute for Global Health Sciences, University of California, San Francisco, CA USA; 2 Center for Malariology, Parasitology and Entomology (CMPE), Ministry of Health, Vientiane, Lao PDR; 3 Global Strategic Information, Institute for Global Health Sciences, University of California, San Francisco, CA USA; 4 Institut Pasteur, Phnom Penh, Cambodia; 5 Malaria Genetic and Resistance Group, Institut Pasteur, Paris, France

## Abstract

**Background:**

Lao People Democratic Republic (PDR; Laos), a landlocked country in Southeast Asia, has made important progress in reducing malaria morbidity and mortality in the past 5–6 years, and the northern provinces have very low reported incidence. To support national progress towards elimination, it is critical to verify and understand these changes in disease burden.

**Methods:**

A two-stage cluster cross-sectional survey was conducted in four districts within four northern provinces (Khua, Phongsaly Province; Paktha, Bokeo Province; Nambak, Luang Prabang, and Muang Et, Huaphanh Province). During September and October 2016, demographics and malaria risk factors were collected from a total of 1492 households. A total of 5085 persons consented to collection of blood samples for testing, by rapid diagnostic test (RDT) and polymerase chain reaction (PCR)-based testing. Risk factors for infection were examined using logistic regression; and a randomized subset of males was tested for glucose-6-phosphate dehydrogenase (G6PD) deficiencies using a combined PCR and sequencing approach.

**Results:**

There were zero positives by RDT, and PCR detected *Plasmodium* infections in 39 (0.77%; 95% CI 0.40–1.47%) of 5082 analysable samples. The species distribution was *Plasmodium vivax* (28 total); *Plasmodium falciparum/P. vivax* (5); *P. falciparum* (3), *Plasmodium malariae* (2), and *P. vivax/P. malariae* (1). In multivariable analysis, the main risk factors included having any other cases within the household [aOR 12.83 (95% CI 4.40 to 37.38), p < 0.001]; and lack of bed net ownership within the household [aOR 10.91 (95% 5.42–21.94), p < 0.001]; age, sex and forest-travel were not associated with parasitaemia. A total of 910 males were tested for the six most common G6PDd in SE Asia; and 30 (3.3%; 95% CI 2.1–5.1%) had a G6PD variant allele associated with G6PD deficiency, with the majority being the Union (14) and Viangchan (11) polymorphisms, with smaller numbers of Canton and Mahidol.

**Conclusion:**

This is the first rigorous PCR-based population survey for malaria infection in Northern Lao PDR, and found a very low prevalence of asymptomatic *Plasmodium* infections by standard PCR methods, with *P. vivax* predominating in the surveyed districts. Clustering of cases within households, and lack of a bed nets suggest reactive case detection, and scale-up of coverage should be prioritized. The predominance of infections with *P. vivax*, combined with moderate levels of serious G6PD deficiencies highlight the need for careful rollout of primaquine towards elimination goals.

**Electronic supplementary material:**

The online version of this article (10.1186/s12936-018-2367-5) contains supplementary material, which is available to authorized users.

## Background

National programmes in the Greater Mekong subregion (GMS) have greatly intensified malaria control and elimination activities in the past decade [1]. Within the region (comprising Cambodia, Lao PDR, Myanmar, Thailand, Vietnam and Yunnan province in China) morbidity has decreased by 74%, and mortality by 91% from 2012 to 2016 [2]. After some setbacks from 2011 to 2015, the Lao People’s Democratic Republic (PDR) is again making rapid progress towards malaria elimination through increased coverage of interventions and intensification of programmatic activities, with an 80% reduction in reported cases from 2010 to 2017 (24,000–9300) [3]. In part due to this progress, the Lao Ministry of Health has made a commitment to eliminate both *Plasmodium falciparum* and *Plasmodium vivax* in northern areas by 2025, followed by elimination of all species nationwide by 2030 [4].

In low transmission settings in Southeast Asia, many malaria infections may remain unidentified by routine passive surveillance within the health sector; several factors contribute to this. These include the sub-clinical parasite reservoir, which is known to make up a sizeable proportion of all infections [5, 6]. Malaria transmission in the GMS is believed to be largely due to occupational exposures (forestry, forest-fringe farming, mining, and a range of semi-legal and illegal activities within forested areas) in these populations who may have limited access to health services [7]. Moreover, these high-risk groups have the potential to transport parasites back into their home communities, thereby perpetuating transmission within villages [8, 9].

While there have been large declines in reported malaria burden specifically in northern provinces within the last several years, these changes have occurred in the absence of any major changes in intervention strategy or coverage by the national malaria programme (CMPE; Center for Malariology, Parasitology and Entomology). The impacts of limitations in data reporting, potential stock outs of commodities, and other changes within the health sector are unknown. It was, therefore, critical to confirm and to contextualize these rapid changes in reported malaria burden to inform national programming.

Understanding factors underpinning the decreasing malaria burden may provide policy guidance for progress in the remainder of the country, and to mitigate the potential for malaria resurgence. To address these knowledge gaps in the malaria epidemiology, a large-scale cross-sectional survey was undertaken to assess malaria parasitaemia and risk factors for infection. This survey was the first of its kind to be undertaken in northern Lao PDR, and provides the only PCR-based survey with rigorous population sampling in the north provinces of the country.

After decades of reporting a majority of infections with *P. falciparum*, the introduction of combination RDTs, and changing epidemiology, *P. vivax* is now a major contribution to malaria burden, comprising 49.2% of all 9300 cases reported nationally in 2017 [3]. To help address this parasite reservoir with safe use of primaquine, a secondary objective of this study was to estimate the prevalence of glucose-6-phosphate dehydrogenase (G6PD) polymorphisms towards elimination of *P. vivax* in these ethnically diverse areas.

## Methods

### Study sites

**Fig. 1:**
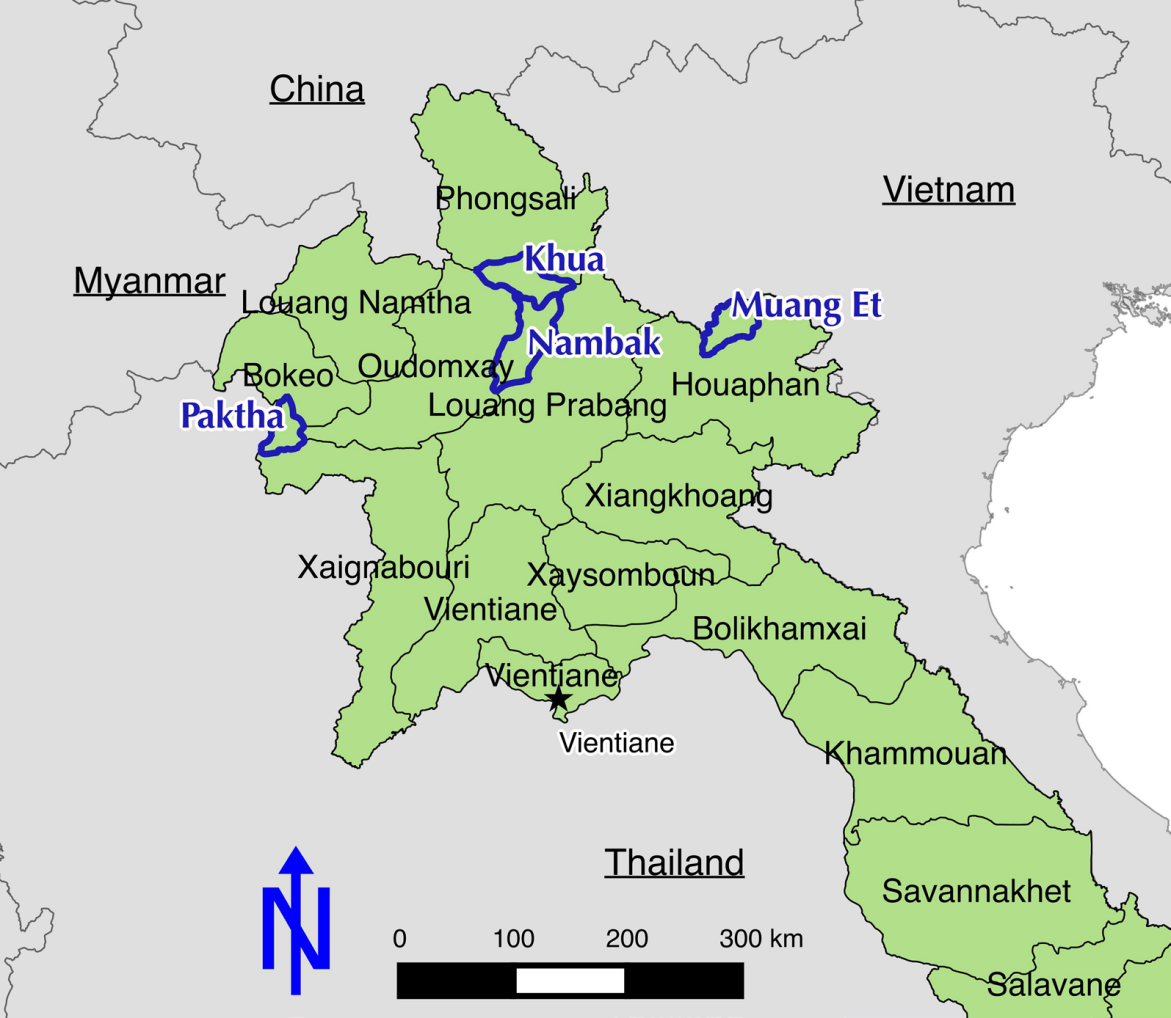
Overview of study sites, Lao PDR

The target four districts (Fig. 1) were chosen in consultation with district- and provincial-level malaria staff focusing on areas with known malaria hotspots, and to capture representative data from diverse epidemiological settings within northern provinces. These provinces are characterized by very mountainous terrain, low population density (as low as 11 per km^2^ in Phongsaly [10]), and diverse climatic zones. The primary malaria vectors include the *Anopheles dirus* and *Anopheles minimus* complexes. *Anopheles dirus* sensu stricto is generally the more efficient vector, but member species of both complexes may be exophagic and exophilic, limiting the impact of standard vector control measures which are currently restricted to ITNs and limited numbers of treated hammock nets [11].

The rainy season generally spans from July through October, and while historically malaria was highly seasonal, there is now less seasonality in reported malaria incidence. This survey was conducted immediately following the rainy season, between 17 September and 20 October 2016. The total population within each district are: Khua, Phongsaly Province (26,164); Paktha, Bokeo Province (19,182); Nambak, Luang Prabang (68,863); and Muang Et, Huaphanh Province (27,001). These are very remote areas, with a total with 21% of residents across the districts residing in villages without road access [10].

### Survey methods

To obtain a representative population sample of the selected districts for the primary outcome of all-species *Plasmodium* prevalence by PCR, a stratified two-stage cluster-sampling design was utilized. Within each district, 25 survey clusters consisting of 50 persons each, were chosen for sampling, to provide 1250 participants per district, and 5000 overall. This sample size was calculated to capture a prevalence of 0.5% with a precision ± 0.2%.

Survey clusters were chosen within each district with probability proportional-to-population size sampling, using preliminary data from the 2015 Lao National Census [10]. Within each cluster, survey staff worked with village leaders to update standard household lists; fifteen houses were then sampled using pre-generated random number lists.

All residents and visitors older than 18 months and present the previous night in these households were invited to participate; after the head of household completed a demographic and risk factor survey (including net ownership/use, any travel, forest exposures, and occupational patterns), a short clinical assessment including axillary temperature, and basic demographics were collected for each consenting household member or visitor (including age, sex, education, primary occupation, and recent travel). Study staff made a maximum of three visits to individual households if all household members were not present. Following written or thumbprint informed consent, all eligible persons were tested with CareStart Ag Pf/Pv (SD Bioline, Cat #05FK80) RDTs and treated as per national guidelines if found positive. Four dried blood spots were also collected on Whatman 903 “Protein Saver” sample cards (GE Healthcare; Cardiff UK), which were dried out of direct sunlight, and stored in cool areas until transfer to refrigerators for subsequent analyses. Geographic coordinates were collected for all participating households, allowing for subsequent mapping of cases and evaluation of clustering at both village and household levels.

All data collection instruments were developed in English, translated into Lao, and back-translated to ensure fidelity by a bilingual heath specialist. These were then pilot-tested and revised before implementation. All field data were collected on paper forms, followed by double-data entry with range checks, by specifically trained staff. Quality assurance (QA) measures included observation of 10% of each enumerator’s household interviews by field team leaders, and recollection of a subset of interview questions by team leaders for 30% of all interviews conducted. Designated QA staff in Vientiane compared the duplicate data collected and flagged any discrepancies to team leaders for immediate follow-up and resolution, including re-interviewing households if required. Upon transfer of data forms to Vientiane weekly, QA staff spot-checked all forms prior to data entry. Data were double-entered using CSPro [12].

### Laboratory methods

For PCR-based testing each dried blood spot was punched with a sterile puncher and placed in a 96-well plate in numerical order. Analyses utilized previously published methods [13]. Samples were lysed overnight in a saponin solution, and DNA was then extracted with Instagen Matrix resin. DNA samples were screened for the presence of *Plasmodium* DNA using a qualitative real-time PCR assay targeting *Plasmodium cytochrome b* gene [13]. Positive samples were then analyzed for *Plasmodium* species using 4 real-time PCR assays specifically amplifying *P. falciparum*, *P. vivax*, *Plasmodium ovale* and *Plasmodium malariae* [13]. PCR followed by Sanger sequencing was performed on all *P. vivax*-positive samples to differentiate any *Plasmodium knowlesi* infections [14].

Based on budget limitations, for G6PD deficiency testing, 910 samples were randomly chosen from all males who provided a blood sample, and tested using PCR-based ligase detection reaction-fluorescent microsphere assays (Luminex; Austin TX, USA) detecting the six most frequent G6PD variants in SE Asia (Mahidol, Mediterranean, Coimbra, Viangchan, Union, and Canton). All detected G6PD variants were then confirmed using a combined PCR and sequencing approach [15].

### Statistical methods

Basic descriptive statistics, including coverage of interventions at household level were calculated with adjustment for survey design. To explore associations with PCR-confirmed parasitaemia and categorical variables, the χ^2^ test was employed; where cells had an expected frequency below five, Fisher’s exact test was used. Associations for continuous variables were examined using the Kruskal–Wallis or Wilcoxon rank-sum test. For each variable, the odds ratio (OR) for malaria infection was calculated using univariate logistic regression. All exploratory variables with biological plausibility as identified in similar studies and p-values ≤ 0.2 were included in multivariable models [16]. A wealth index for households was constructed from household assets using principle components analysis [17].

Akaike and Bayesian Information Criterion (AIC/BIC) were both used to compare model fit; all reported models were adjusted for age, sex, and district. Collinearities and interactions were assessed, and where collinearities were identified, variables were retained based on the comparison of AIC/BIC from models with inclusion of individual variables. After identification of a preliminary refined model, residuals and leverage metrics were examined, and while a small population of highly influential observations was identified, this included several PCR-positives, and so these were retained for analysis. Final reported models were checked with the Archer-Lemeshow goodness-of-fit test (by deciles) [14].

All statistical tests were two-tailed with α = 0.05, and all reported proportions are adjusted for survey design effects. All statistical analyses were conducted using Stata 14.1 (College Station, Texas, US), and geographic visualizations were conducted using QGIS software [18].

### Model specification-logistic regressions

The limited number of parasitaemia positives within this survey presented some challenges in analysis. These issues, which have been well-documented in the area of rare event regressions, involve sparse strata and potentially biased estimates in maximum likelihood estimation when events are below about 1% of total persons or events captured [19]. To mitigate these issues and assess the robustness of results and subsequent conclusions, multiple model specifications were explored in this analysis.

The model specifications included logistic regression with adjustments for survey design (Stata, *svy: logistic*); multilevel models with random effects for the village-level and household-level and survey design (Stata, *svy: melogit*); and penalized likelihood bias-reduction logistic regression (Stata, *firthlogit* [20]). The Firth penalized-likelihood method has been proposed for bias reduction in rare-event logistic regressions in complex survey samples, but, this approach has not been fully validated [21]. Mixed models (with both village and village + household level random effects) were assessed but either did not converge using a range of maximizations, or show improvement of model fit, and were not pursued.

## Results

### Study population

**Fig. 2:**
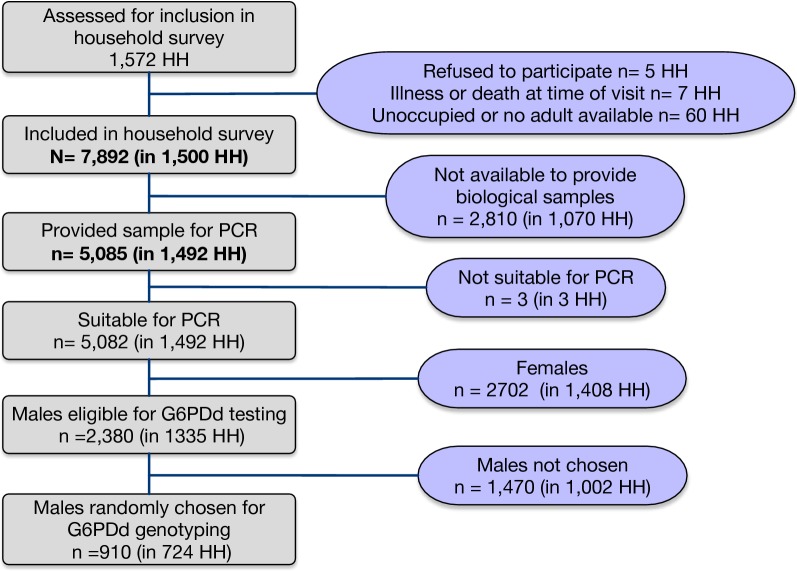
CONSORT diagram of study enrollment

A total of 7892 persons were reported as residents within 1500 households. Of these, 5085 persons in 1492 households consented to collection of blood samples for testing; Fig. 2 provides an overview of study enrollment.

In comparison to the total population of surveyed individuals, those that provided biological samples were more likely to be female (bio-sampled: 53.2% female vs. overall household population: 45.0%; Fisher’s exact test for difference, p < 0.0001), and were older (mean age, bio-sampled: 29.5. vs. 20.3 for overall survey; t-test for differences, p < 0.0001).

Primary occupations also differed between those tested and those not available for testing (χ^2^ test; p < 0.001); the proportion of plantation workers and small-scale farmers was higher in those who had parasitological testing, whereas the proportion of students was higher in those not present for testing. Selected population characteristics can be found in Table 1; more detailed demographic characteristics can be found in Additional file 1.

**Table 1:** Selected demographic features of survey participants (N = 5082), malaria parasite and risk-factor survey, Northern Lao PDR

Characteristic	n	% of total (95% CI)
Sex		
Male	2380	46.8 (45.7–48.0%)
Female	2702	53.2 (52.0–54.3%)
Age group		
< 5	273	5.4 (4.6–6.3%)
5–15	1198	23.6 (21.9–25.3%)
> 15	3611	71.0 (73.0–36.1%)
Slept under treated net previous night		
Yes	1767	34.8 (29.0–41.0%)
No	3315	65.2 (59.0–71.0%)
Slept under any net previous night		
Yes	1781	35.1 (29.3–41.3%)
No	3301	65.0 (58.7–70.7%)
Febrile at survey (≥ 37.5 °C)		
Yes	22	0.43 (0.27–0.7%)
No	5057	99.6 (99.3–99.7%)
Age, if febrile at survey (≥ 37.5 °C)		median (IQR): 25.5 (9–35)
Resident in household with any fever in 2 weeks prior to survey		
Yes	1144	22.5 (19.2–26.2%)
No	3398	77.5 (73.8–80.1%)
Resident in household with any forest-goers		
Yes	1910	37.6 (32.0–43.5%)
No	3172	62.4 (56.5–68.0%)

“Forest-goer” defined as household where anyone sometimes go to the forest, forest fringe, farms, or rice fields and sleeps there overnight

### Sample descriptive statistics

A total of 5085 persons consented to collection of blood samples for testing, and there were zero RDT-positives within the survey. Three of the DBS samples were unsuitable for analysis, leaving a total of 5082 for PCR-based-parasitaemia testing. A total of 39 persons were positive by PCR, for an overall prevalence for all species of 0.77% (95% CI 0.40–1.47%). The majority of cases (28) were mono-infection with *P. vivax*, with cases found in a total of 31 households (2.7%; 95% CI 1.6–4.5%) (Table 2). Of these, 26 households had a single parasitaemic individual; three households had two parasitaemic persons; and single households were found with three and four parasitaemic individuals respectively.

**Table 2:** Malaria parasite species identified by PCR (N = 39), Northern Lao PDR

Species	n	% of PCR-positives; 95% CI
*Plasmodium vivax*	28	71.8 (46.1–88.4)
*P. falciparum/P. vivax*	5	12.8 (4.5–31.6)
*P. falciparum*	3	7.7 (1.9–26.7)
*P. malariae*	2	5.1 (1.1–21.6)
*P. vivax/P. malariae*	1	2.6 (0.3–18.7)
All species	39	

The reported main occupations were consistent with the rural population: 50.6% reported being small-scale farmers. Of the 1492 households sampled, 35.8% reported that at least one household member sometimes slept overnight at forested, forest-fringe or rice-field sites; and 13.9% of all 1492 households reported household members travelling outside the province within the previous 12 months. Of these, 46.8% were male with a median age of 27 years (IQR 14–42).

Bed net ownership was nearly universal, with 99.5% of 1492 households reported owning at least one net of any type. However, only 70.5% of households reported owning at least one pre-treated net, and a slight majority (53.0%) of households had at least one treated net for every two residents. A total of 22 people (0.43%; 95% CI 95% CI 0.27–0.7) had a fever (temperature ≥ 37.5 °C) at survey; 21.0% of households reported having any fever within previous 2 weeks; and five households (0.33%) reported having a parasitologically-confirmed malaria case within the 2-week period prior to survey.

### G6PD variants detection

A total of 30 out of 910 samples sequenced from males (3.5%; 95% CI 2.2–5.4%) had a G6PD variant allele associated with G6PD deficiency (Table 3), with the majority being the Union (14) and Viangchan (11) polymorphisms, with smaller numbers of Canton and Mahidol. The Mediterranean and Coimbra variants were not found in the survey. Prevalence in tested males ranged from 0.6% (95% CI 0.08–4.4%) in Paktha to 6.0% (95% CI 3.0–11.4%) in Muang Et. There was a significant difference in proportions between the study sites (design-based F-test, p = 0.0089).

**Table 3:** Glucose-6-phosphate dehydrogenase (G6PD) variants identified by sequencing after initial Luminex screen, Northern Lao PDR (N = 910)

Genotype	n	% of total tested (95% CI)
Union	14	1.5 (0.84–2.6)
Viangchan	11	1.2 (0.60–2.2)
Canton	4	0.44 (0.12–1.1)
Mahidol	1	0.11 (0.028–0.61)
Wild type	878	96.5 (95.1–97.6)
Not interpretable	2	0.22 (0.027–0.80)

While this survey was not powered to assess any association between G6PDd and PCR-based positivity, a total of nine PCR-positives were also tested for G6PDd (seven *P. vivax*; one *P. falciparum/P. vivax* mixed; and one *P. malariae*). All were found to be G6PD normal (G6PD wild-type alleles).

### Bivariate analysis

**Fig. 3:**
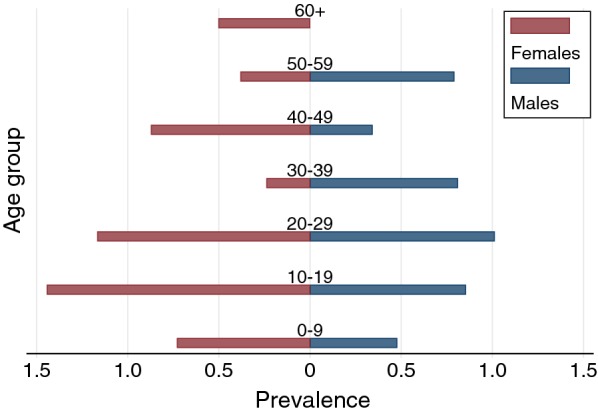
Age-stratified malaria prevalence rate (all species) by PCR, Northern Lao PDR

There was no association between fever and PCR-positivity; all PCR-positives were afebrile at survey (with fever defined as ≥ 37.5 °C), and there was no difference in mean axillary temperature between the two groups (t-test for differences in means; p = 0.215). Cases were found in all age groups (range 4–65; median 21, mean 24.8); 41% were male and 59% female (Fig. 3).

Among the non-vivax cases, three of the *P. falciparum/P. vivax* mixed infections reported individual- or household-level travel outside the province within the previous 12 months, while the remainder of non-vivax cases (n = 8) did not report any travel by household members, and included both students and retirees.

### Care-seeking and drug therapy

The care-seeking cascade for all households that reported any febrile illness in the 2 weeks prior to the survey was examined; there was also no association between household-level PCR-positivity and any fever in the household within the past 2 weeks (χ^2^ test, p = 0.268); see Additional file 2 for this cascade. Several important gaps in early diagnosis and treatment were apparent. Of the 286 persons (75.1% of those reporting a fever) who sought care outside the household, 94 (32.9%) waited 2 days or more before seeking care. Of those who did not seek care (n = 77), the main reasons were reported (with multiple answers possible) to be simply waiting for the fever to go away (n = 16; 20.8%), and not having money for treatment (n = 36; 46.8%).

While the majority of respondents reported seeking care in a public facility at any point (n = 259; 90.6%), only a small minority of these reported receiving RDT-based testing (n = 25; 9.7%; with no differences by age or sex, via χ^2^ test). Of the 234 who reported not receiving an RDT-based test, one was positive by PCR at survey (*P. vivax*; a 4-year old male). Of those that reported being tested by RDT, five were found positive, representing a test positivity rate of 20%. Finally, of these five positives (one *P. vivax*; the remainder were unable to provide the species), only two were treated as per national guidelines with any regimen of artemisinin-based combination therapy (ACT) (one reported being prescribed the standard of care in Lao PDR (artemether-lumefantrine), with the other reported as ‘any other ACT’; one didn’t know; and two were given aspirin and/or paracetamol only).

### Multivariable regression

**Fig. 4:**
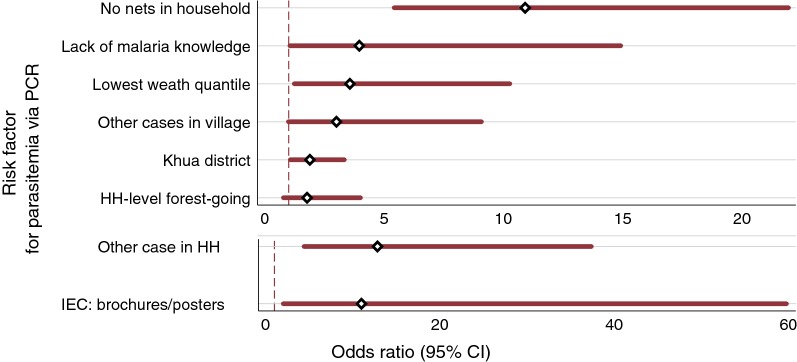
Adjusted odds ratios, associations with PCR-based parasitaemia (all species), Northern Lao PDR. *HH* household

As few samples were positive by PCR-based testing, risk factors for all *Plasmodium* species were combined into a single outcome of ‘any parasitaemia’ for both univariate and multivariable analyses. Figure 4 gives an overview of all identified risk factors for sub-patent parasitaemia from multivariable logistic models adjusted for survey design, sex and age; full results can be found in Table 4. Results from Firth penalized likelihood models can be found in Additional file 3.

**Table 4:** Multivariable risk factors for PCR-positivity (all species), malaria parasite and risk-factor survey, Northern Lao PDR

Risk factor		Unadjusted OR	[95% CI]	p-value	aOR	[95% CI]	p-value
District	Paktha	Reference	–	–	Reference	–	–
	Muang Et	0.96	0.31–2.94	0.942	1.77	0.74–4.24	0.197
	Nambak	0.28	0.035–2.28	0.232	0.28	0.048–1.64	0.157
	Khua	2.91	0.83–10.18	0.94	*1.89*	*1.07–3.34*	*0.029*
Wealth quantile	Highest	Reference	–	–	Reference	–	–
	Middle high	1.95	0.57–6.60	0.281	2.13	0.87–5.24	0.097
	Middle low	2.03	0.63–6.46	0.229	1.63	0.59–4.50	0.345
	Lowest	*6.78*	*1.84*–*24.93*	*0.004*	*3.57*	*1.24–10.27*	*0.019*
Does HH own any type of net (bed net or hammock) to sleep under?	Yes	Reference	–	–	Reference	–	
	No	*31.90*	*4.35–234.12*	*0.001*	*10.91*	*5.42–21.94*	*< 0.001*
Have you ever heard of malaria?	Yes	Reference	–	–	Reference	–	–
	No	1.44	0.61–3.39	0.396	0.71	0.38–1.34	0.286
	Don’t know	*5.05*	*1.77–3.39*	*0.003*	*3.96*	*1.05–14.91*	*0.042*
Source of malaria knowledge	Not brochure or poster	Reference	–	–	Reference	–	–
	Brochure or poster	5.28	0.637–44.46	0.124	*10.99*	*2.02–59.77*	*0.006*
Does anyone in HH go to forest or forest-fringe sites overnight?	No	Reference	–	–	Reference	–	–
	Yes	*3.35*	*1.10–10.21*	*0.033*	1.77	0.78–4.01	0.169
Any cases in village outside household?	No	Reference	–	–	Reference	–	–
	Yes	2.07	0.13–5.30	0.128	3.00	0.993–9.08	0.051
Any other cases in household?	No	Reference	–	–	Reference	–	–
	Yes	*25.77*	*7.52–88.31*	*< 0.001*	*12.83*	*4.40–37.38*	*< 0.001*
Sex	Male	Reference	–	–	Reference		–
	Female	1.27	0.80–2.00	0.305	1.18	0.69–2.02	0.549
Age in years	Continuous	0.986	0.97–1.01	0.146	0.995	0.98–1.01	0.563

Risk factors with p < 0.05 are shown in italicface

*HH* household

The strongest predictor for parasitaemia was the presence of any other parasitaemic individuals within the household [Adjusted odds ratio (aOR) = 12.83, 95% CI 4.40–37.38; p < 0.001]. This odds ratio is more than seven times what would be expected if infections were randomly allocated (OR = 1.8; see Additional file 4) indicating a high degree of household-level clustering. There was also a strong association with the lack of net ownership (any type) within the household, (aOR = 10.91, 95% CI 5.42–21.94; p < 0.001); there was no association with household-level ownership of any treated nets.

Geographic location and socioeconomic factors were also statistically significant, and residents of Khua district had elevated risk of parasitaemia relative to residents of Paktha (aOR = 1.89, 95% CI 1.07–3.34; p = 0.029). Persons living within a household in the lowest wealth quartile, relative to the highest quartile, had increased risk for parasitaemia (aOR = 3.57, 95% CI 1.24–10.27; p = 0.019), which was attenuated from the univariate estimate (OR = 6.78, 95% CI 1.84–24.93; p = 0.004). While not significant in univariate models (OR = 5.28, 95% CI 0.63–44.46; p = 0.124), naming exposure to brochures or posters as a source of malaria knowledge was significant after covariate adjustment (aOR = 10.99, 95% CI 2.02–59.77; p = 0.042). One other factor showed marginal significance: having limited malaria knowledge (“Don’t know if ever have heard of malaria”) relative to having malaria knowledge (aOR = 3.69, 95% CI 1.05, 14.91, p = 0.042), while any cases within the village not in the same household (aOR 3.00, 95% CI 0.993–9.08, p = 0.51) was not significant.

Age and sex were not significantly associated with parasitaemia in either univariate or adjusted models. While there was a significant association between parasitaemia and forest-going activities by any member of the household in univariate analysis (OR = 3.35, 95% CI 1.10–10.21; p = 0.033), this was no longer a significant factor in the fully-adjusted model (aOR = 1.77, 95% CI 0.78–4.01; p = 0.169), potentially due to adjustment for wealth index.

Results and conclusions with analysis restricted to *P. vivax* mono-infections as the outcome (n = 28 PCR-positives; N = 5071) were largely consistent with overall results, with some important exceptions. Any forest-going by household members was a significant predictor of *P. vivax* infection, with an aOR of 3.40 (95% CI 1.26–9.21; p = 0.016); and the presence of any other village-level cases was also significant, with an aOR of 4.93 (1.30–18.62; p = 0.019); age and sex remained not significant. Finally, presence of other cases within the household showed an increased association with parasitaemia with an aOR of 21.82 (6.40–74.40; p < 0.001). This odds ratio also represents a biased estimate due to the low prevalence and should be interpreted with caution. This odds ratio is more than 12-fold higher what would be expected if infections were randomly allocated (OR = 1.8; see Additional file 4) again, indicating a high degree of household-level clustering. Finally, contingency tables were used to further explore the relationship between infecting parasite species and travel (either personally, or within the household) both outside the province and outside Lao PDR within 12 months, but no associations were significant (Fisher’s exact test; all p > 0.257).

## Discussion

In this study, the first of its kind in Northern Lao PDR, low population-level parasitaemia was found, in addition to moderate levels of G6PDd. The main risk factors for malaria parasitaemia in four districts of Northern Lao PDR studied here are other cases within households, lack of any type of nets, and socio-economic status, while forest-going, sex and age were not significant.

However, this lack of associations may be due to limited village-based transmission, or to the low prevalence, which inherently limits the power for determining associations (these association were all powered at < 0.5). In contrast, the finding of an association between forest-going and *P. vivax* mono-infections in this current study may be explained by field data and modeling which both suggest that the majority of *P. vivax* infections are due to relapses from dormant hypnozoites and not new infections [22, 23] and if, as has been suggested, that anopheline exposure itself may trigger relapses [24].

While previously identified in Lao PDR [25] the *P. malariae* infections in this survey have important implications, as the long extrinsic incubation period for this species implies the existence of long-lived vectors [26, 27] in surveyed areas. While not specifically powered to discover any, no *P. knowlesi* infections were identified in this survey. Human infections with *P. knowlesi* have been reported throughout the other GMS countries, but the species has only been reported in a single human sample from southern Lao PDR [28].

The increased risk of malaria from lack of any type of bed nets within households in this work is somewhat unexpected within the GMS, where effectiveness is limited or equivocal [29]. Proposed reasons [11] for limited impact in the region are from exophagic and exophilic vector complexes, and shifts in peak biting times to periods when net usage is low (evenings and early morning) [30]. Recent entomological surveys in Northern Lao PDR found higher exposure to *Anopheles* spp. in villages relative to secondary forests (OR = 1.95, 95% CI 1.60–2.39; p < 0.001), but with important differences by landscape type when examined by specific vector species [31]. The increased risk for parasitaemia in households where poster- or brochure-based health materials were found (as opposed to those who did not receive these materials), is surprising, but may be due to well-targeted interventions to highest-risk areas, or potentially limitations in the applicability of general county-wide IEC/BCC materials in populations with high ethnic diversity [32]. Further work should be undertaken to ensure materials are well-aligned with malaria knowledge gaps.

PCR-based testing from samples collected in 2013 in southern Lao PDR (Savannakhet province) found predominately *P. falciparum* infections. In that setting, age, sex, and forest-based exposures were not associated with parasitaemia, and the only significant predictor for parasitaemia (all species) was the presence of any other cases within the household, with an aOR of 24.33 (95% CI 10.15–58.32; p < 0.001) [33]. This estimate is consistent with that in the present study, suggesting that clustering of cases within households is an important and consistent epidemiological feature in many settings throughout Lao PDR. In contrast, a recent survey in 2015 in southern Lao PDR [34] found that parasitaemia was strongly associated with working-age males in multivariable analysis for *P. vivax* mono-infections, but neither age nor sex were significant for *P. falciparum* mono-infections. Overnight stays in the forest within the previous 3 months were also associated with being parasitaemic with *P. vivax*, but not for *P. falciparum.* This divergent epidemiology between the two species has also been identified in other settings, including Cambodia, Indonesia, and Papua New Guinea [35–37].

The very low prevalence of fever in this survey (0.43%; 95% CI 0.27–0.7; N = 5082) is consistent with other recent studies with Lao PDR. In Savannakhet, a higher-burden province, there were two febrile persons (≥ 37.5 °C) among 888 participants (0.23%; 95% CI 0.027–0.81%) [34]. These findings and the lack of RDT-positives in this survey reinforce earlier studies suggesting most malaria infections in Lao PDR are sub-clinical and below the limit of detection for standard RDTs [34]. High sensitivity RDTs for *P. vivax* (currently in development), and field-based platforms (e.g., loop-mediated isothermal amplification method, LAMP [38]) should be considered to address this surveillance gap [6].

There are limited data on G6PD deficiencies in Lao PDR, and the moderate levels of G6PD variants associated to G6PDd in males, consistent with other areas in the GMS [39], will still require careful implementation of G6PD testing for safe use of primaquine. The high prevalence of Mahidol and Viangchan variants will be important for full-course primaquine in the treatment of *P. vivax*. While earlier schemes listed these variants as only moderate deficiencies (WHO class II or III), this classification may be problematic [40], and these patients could be at high risk for serious G6PD-related hemolysis [41]. Finally, while overall numbers tested are small, the wide variation of G6PDd prevalence in surveyed districts highlights the critical importance of local testing to ensure safe use of primaquine. Moreover, there is potential for other G6PDd variants to be present in Northern Lao PDR, as only a limited subset of known variants was tested for.

This study suggests that malaria testing rates in public facilities may be sub-optimal [3]. Testing rates at facilities should be greatly increased through ensuring full stocks of RDTs, refresher training for health staff to test all febrile illness, and BCC/IEC campaigns to support early diagnosis and treatment. While this survey itself did not query providers on adherence to testing algorithms, formative work and facility visits suggest that stock-outs are a worry for staff, who may ‘triage’ patients for testing, to conserve both RDTs and ACTs for other patients. However, the extent and frequency of this is not known. The Lao National Center for Malariology, Parasitology and Entomology has recently (late 2017) updated all facility-level materials for clinical care and surveillance, and more research should be undertaken to examine the impacts of these materials and associated trainings. Finally, population-level blood annual examination rates should be aligned with the 10% guidance target used in historical malaria programmes [42].

While RDT-based testing and subsequent treatment are free at all public health facilities in Lao PDR, the costs associated with care seeking were reported in this survey to be important barriers to early diagnosis and treatment. Delays in care-seeking of even a single day may provide opportunities for onward transmission of *P. vivax*, where gametocytes are present in the earliest stages of infection [43]. Indeed, one respondent in this survey (a 10-year old female), who reported not seeking care due to lack of funds had an asymptomatic *P. vivax* infection at survey, as did another member of the same household.

Taken in summary, the findings from this survey suggest that expansion of several programmatic activities could have important epidemiological impacts. Specifically, case-based surveillance and response strategies should be rapidly implemented, and are especially feasible given the very low malaria case burden. To target vectors and sub-clinical malaria cases within households, a locally-appropriate variation of China’s “1,3,7” programme, aligned with the difficult terrain and access in Northern Lao PDR, should be considered [44]. Moreover, while not efficient in all settings [45], reactive case detection (RACD) and response may have an important role in northern provinces; to date there has been limited trialing of RACD in Lao PDR overall.

This study is not without limitations. Firstly, the sampled districts may not be fully representative of all transmission settings in northern areas or within other provinces in northern Lao PDR. Important variation may exist, especially in light of the low prevalence by PCR. Secondly, although there were strenuous efforts to capture blood samples from all enumerated household members, some age groups were underrepresented, and could be parasite reservoirs in this population. Beyond producing wide confidence intervals for odds ratios, models based upon the limited number of PCR-confirmed infections also have the potential to produce biased point estimates [46], but the broad consistency between models suggests this is of limited impact. Finally, the outcome of “any other cases in the household” may be biased at very low prevalence, (Additional file 4). While this is not a formal test of spatial aggregation, these effect sizes for the outcomes of all-species and *P. vivax*-only parasitaemia each represent substantial evidence for clustering of cases within households, as cases are overdispersed [47].

## Conclusions

The Northern provinces of Lao PDR have made important progress in reducing the incidence of all malaria species in the past decade, but some areas of continued transmission remain, which will require targeted interventions towards sub-national elimination targets. The low parasite burden highlights the rapid feasibility of sub-national elimination, but some programmatic challenges remain.

Targeting of interventions to highest-risk groups is essential as malaria burdens decrease to maximize the impact of inherently limited funding within the health sector. The findings from this study suggest that assumptions about adult males with forest- or field-based work exposures being the primary risk group should be reexamined, as cases were found equally in all demographics.

Moreover, reactive case detection focused on household members of an index case may be a useful strategy, especially using sensitive analytical methods or mass test and treat of household contacts. Potential gaps in early diagnosis and treatment suggest that successful programming will also require addressing delays in care-seeking, including low reported facility-level testing rates, and towards increased net coverage. These in turn will require expansion of supportive supervision at provincial- and district-levels to promote consistent implementation. Attacking the substantial vivax hypnozoite reservoir will require routine G6PDd testing in a population having moderate levels of deficiencies, followed by consistent and complete primaquine treatment. In this context, the benefits of well-support village health workers should be considered in addressing many identified gaps in early diagnosis and treatment within northern Lao PDR for accelerated progress [48, 49].

## Additional files

**Additional file 1.** Detailed population characteristics, malaria parasite and risk-factor survey, Northern Lao PDR (N= 5,082).

**Additional file 2.** Malaria early diagnosis and treatment (EDAT) cascade for self-reported febrile illness, Northern Lao PDR.

**Additional file 3.** Multivariable Firth penalized-likelihood logistic model for PCR-based malaria parasite positivity (all species), Northern Lao PDR (N= 5,082).

**Additional file 4.** Sensitivity analysis for the odds ratios, risk of parasitaemia with “any other case in household.”
