# Supplementary material for: Prevalence and risk factors for asymptomatic malaria and genotyping of glucose 6-phosphate (G6PD) deficiencies in a vivax-predominant setting, Lao PDR: implications for sub-national elimination goals
Source: Malar J. 2018 Jun 1;17:218. doi: 10.1186/s12936-018-2367-5 (PMC5984820; doi:10.1186/s12936-018-2367-5)
Supplement: Supplementary file 1 — Additional file 1. Detailed population characteristics, malaria parasite and risk-factor survey, Northern Lao PDR (N= 5,082). [file 12936_2018_2367_MOESM1_ESM.pdf]

Additional file 1.

Detailed population characteristics, malaria parasite and risk-factor survey, Northern Lao PDR (N= 5,082) (Part 1).

characteristic		n	% of total (95% CI)
**Sex**	male	2,380	46.8 (45.7 to 48.0)
	female	2,702	53.2 (52.0 to 54.3)
**Age**	0-9	830	16.3 (14.8 to 18.0)
	10-19	1,022	20.1 (18.6 to 21.7)
	20-29	909	17.9 (16.6 to 19.2)
	30-39	788	15.5 (14.4 to 16.6)
	40-49	636	12.5 (11.4 to 13.7)
	50-59	515	10.1 (9.2 to 11.2)
	60+	382	7.5 (6.6 to 8.6)
**District**	Paktha	1,038	20.4 (13.9 to 29.0)
	Muang Et	1,442	28.4 (19.9 to 38.8)
	Nambak	1,219	24.0 (16.5 to 33.6)
	Khua	1,383	27.2 (19.0 to 37.4)
**Education**	No schooling	1,102	21.7 (19.2 to 24.4)
	Some primary	1,996	39.3 (37.3 to 41.3)
	Completed primary (grade 5)	709	14.0 (12.5 to 15.6)
	Some secondary	896	17.6 (15.4 to 20.1)
	Completed secondary (grade 11 or 12)	267	5.3 (4.2 to 6.6)
	More than secondary	112	2.2 (1.7 to 2.9)
**Primary occupation**	Small-scale farmer	2,014	39.6 (37.4 to 41.9)
	Student	1,391	
	Not of working age/homemaker	1,102	21.7 (19.2 to 24.4)
	Manual labor	216	4.3 (3.2 to 5.6)
	Plantation work (for cash)	130	2.6 (1.8 to 3.6)
	Small business owner	84	1.7 (1.1 to 2.5)
	Not working	81	1.6 (1.2 to 2.1)
	Teacher	58	1,1 (0.80 to 1.6)
	Collecting plants or hunting in forest	6	0.12 (0.047 to 0.30)
**Household residency status**	Visitor	10	0.20 (0.087 to 0.44)

	Resident	5,072	99.8 (99.6 to 99.9)
**Wealth quintile**	Highest	1,126	22.2 (18.2 to 26.7)
	Middle high	1,238	24.4 (20.9 to 28.2)
	Middle low	1,288	25.3 (21.9 to 29.1)
	Lowest	1,430	28.1 (22.0 to 35.2)
**Resident of household with ownership of bednet (any type)**	No	22	0.43 (0.013 to 0.15)
	Yes	5,060	99.6 (98.5 to 99.9)
**Resident of household with at least one treated bednet for every two residents**	No	2,368	46.6 (40.5 to 52.8)
	Yes	2,692	53.0 (46.7 to 59.2)

Detailed population characteristics, malaria parasite and risk-factor survey, Northern Lao PDR (N= 5,082) (Part 2).

**characteristic**		**n**	**% of total (95% CI)**
**Resident of household with any forest-goers**	No	3,172	62.4 (56.5 to 68.0)
	Yes	1,970	37.6 (32.0 to 43.5)
**Resident of household where source of malaria information is brochures or posters**	No	5,056	99.5 (98.8 to 99.8)
**Have you ever heard of malaria?**	Yes	3,702	72.9 (68.9 to 76.5)
	No	1,286	25.3 (21.6 to 29.4)
	Don’t know	94	1.9 (1.2 to 2.8)
**Presence of other PCR-positives in village, but not in household**	No	4,178	82.2 (73.8 to 88.4)

	Yes	904	17.8 (11.6 to 26.2)
**Presence of other cases in household**	No	4,973	97.9 (96.2 to 98.8)
	Yes	109	2.1 (1.2 to 3.8)
**Mosquito nets (any type) per household**	mean	-	4.3 (4.2 to 4.5)
	range	-	1 to 14
	person per net ratio	-	1.5 (1.4 to 1.6)
**Treated mosquito nets (any type) per household**	mean	-	2.6
	range	-	0 to 12
	person per net ratio	-	1.9 (1.7 to 2.0)
