# Supplementary material for: Prevalence and risk factors for asymptomatic malaria and genotyping of glucose 6-phosphate (G6PD) deficiencies in a vivax-predominant setting, Lao PDR: implications for sub-national elimination goals
Source: Malar J. 2018 Jun 1;17:218. doi: 10.1186/s12936-018-2367-5 (PMC5984820; doi:10.1186/s12936-018-2367-5)
Supplement: Supplementary file 2 — Additional file 2. Malaria early diagnosis and treatment (EDAT) cascade for self-reported febrile illness, Northern Lao PDR. [file 12936_2018_2367_MOESM2_ESM.pdf]

Additional file 2.

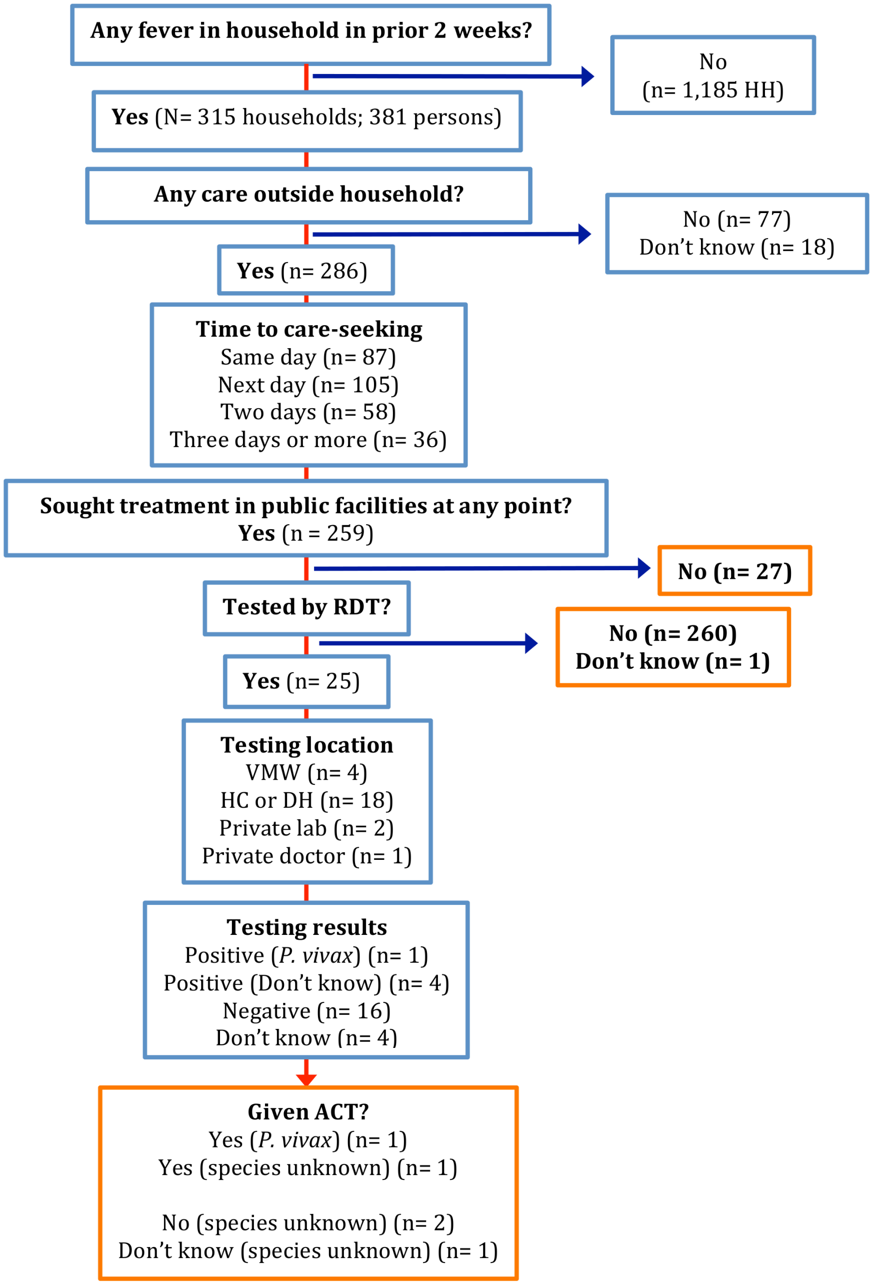

Additional file 1. Malaria early diagnosis and treatment (EDAT) cascade for self-reported febrile illness, Northern Lao PDR.

Notes: HC= health center; DH= district hospital; VMW= village health/malaria worker; ACT= artemisinin-based combination therapy.
