# Supplementary material for: Prevalence and risk factors for asymptomatic malaria and genotyping of glucose 6-phosphate (G6PD) deficiencies in a vivax-predominant setting, Lao PDR: implications for sub-national elimination goals
Source: Malar J. 2018 Jun 1;17:218. doi: 10.1186/s12936-018-2367-5 (PMC5984820; doi:10.1186/s12936-018-2367-5)
Supplement: Supplementary file 3 — Additional file 3. Multivariable Firth penalized-likelihood logistic model for PCR-based malaria parasite positivity (all species), Northern Lao PDR (N= 5,082). [file 12936_2018_2367_MOESM3_ESM.pdf]

Additional file 3:. Multivariable Firth penalized-likelihood logistic model for PCR-based malaria parasite positivity (all species), Northern Lao PDR (N= 5,082).
Note: risk factors with p < 0.05 are in boldface; HH= household.

Risk factor		aOR	[95% Conf. Interval]	p-value
District	Paktha	reference	-	-
	Muang Et	1.70	0.51 to 5.65	0.386
	Nambak	0.33	0.070 to 1.61	0.171
	Khua	1.74	0.63 to 4.75	0.282
Wealth quantile	highest	reference	-	-
	middle high	2.11	0.58 to 7.61	0.254
	middle low	1.59	0.41 to 6.11	0.499
	lowest	3.38	0.95 to 12.00	0.060
Does HH own any type of net (bednet or hammock) to sleep under?	Yes	reference	-	-
	**No**	**10.32**	**2.07 to 51.53**	**0.004**
Have you ever heard of malaria?	Yes	reference	-	-
	No	0.73	0.32 to 1.65	0.447
	**Don't know**	**4.21**	**1.06 to 16.78**	**0.041**
Source of malaria knowledge	Not brochure or poster	reference	-	-
	**Brochure or poster**	**14.05**	**2.04 to 96.96**	**0.007**
Does anyone in HH go to forest or forest-fringe sites overnight?	No	reference	-	-
	Yes	1.74	0.71 to 4.26	0.222
Any cases in village outside household?	No	reference	-	-
	**Yes**	**2.99**	**1.37 to 6.52**	**0.006**
Any other cases in household?	No	reference	-	-
	**Yes**	**12.04**	**4.97 to 29.19**	**< 0.001**
Sex	male	reference	-	-
	female	1.18	0.61 to 2.27	0.632
Age in years	continuous	0.996	0.98 to 1.01	0.652
