# Supplementary material for: Prevalence and risk factors for asymptomatic malaria and genotyping of glucose 6-phosphate (G6PD) deficiencies in a vivax-predominant setting, Lao PDR: implications for sub-national elimination goals
Source: Malar J. 2018 Jun 1;17:218. doi: 10.1186/s12936-018-2367-5 (PMC5984820; doi:10.1186/s12936-018-2367-5)
Supplement: Supplementary file 4 — Additional file 4. Sensitivity analysis for the odds ratios, risk of parasitaemia with “any other case in household.” [file 12936_2018_2367_MOESM4_ESM.pdf]

Additional File 4. Sensitivity analysis for the odds ratios, risk of parasitemia with "any other case in household"

Methods. To assess the potential role of random household-level aggregation of cases in influencing the observed odds ratios for "any other cases within a household," simulations were performed where a given prevalence was assigned as a random binomial to the entire survey population (N= 5,082). Using the surveyed household structure, logistic regression was then used to obtain odds ratios for parasitemia, with adjustment for household size. A set of 2,000 simulations was perfomred for each assigned prevalence; reported confidence intervals are binomial exact. All analyses used Stata 15.1 (College Station TX, USA) with 95% CIs.

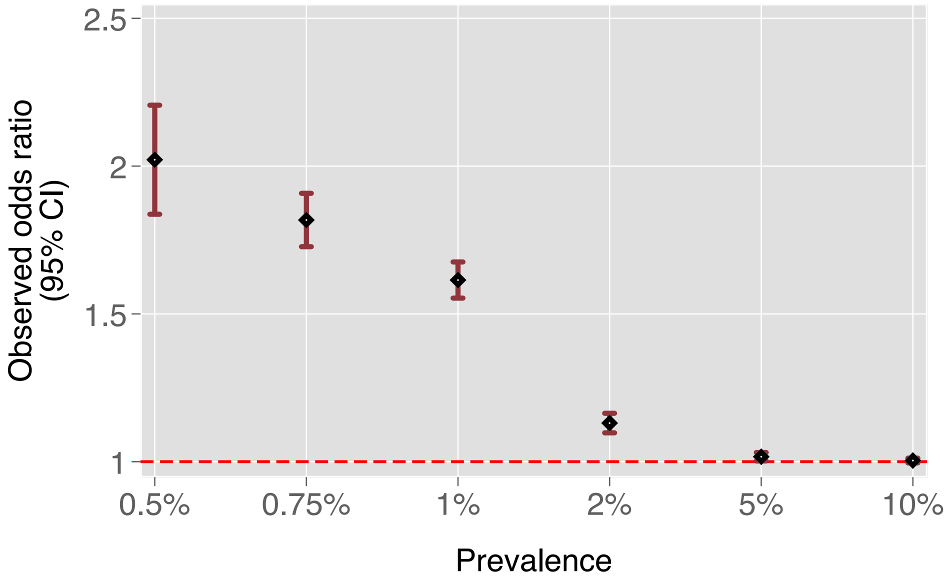

Figure. Sensitivity analysis for the odds ratios, risk of parasitemia with "any other case in household" with randomized assignment of indicated prevalences (notes: x-axis not to scale; see text for details).
